# *N*-(3-Chloro-4-eth­oxy-1-methyl-1*H*-indazol-5-yl)-4-meth­oxy­benzene­sulfonamide

**DOI:** 10.1107/S1600536814010800

**Published:** 2014-05-17

**Authors:** Hakima Chicha, El Mostapha Rakib, Abdellah Hannioui, Mohamed Saadi, Lahcen El Ammari

**Affiliations:** a Laboratoire de Chimie Organique et Analytique, Université Sultan Moulay Slimane, Faculté des Sciences et Techniques, Béni-Mellal, BP 523, Morocco; b Laboratoire de Chimie du Solide Appliquée, Faculté des Sciences, Université Mohammed V-Agdal, Avenue Ibn Battouta, BP. 1014, Rabat, Morocco

## Abstract

The indazole ring system of the title compound, C_17_H_18_ClN_3_O_4_S, is almost planar (r.m.s. deviation = 0.0113 Å) and forms dihedral angles of 32.22 (8) and 57.5 (3)° with the benzene ring and the mean plane through the 4-eth­oxy group, respectively. In the crystal, mol­ecules are connected by pairs of N—H⋯O hydrogen bonds into inversion dimers, which are further linked by π–π inter­actions between the diazole rings [inter­centroid distance = 3.4946 (11) Å], forming chains parallel to [101].

## Related literature   

For the biological activity of sulfonamides, see: El-Sayed *et al.* (2011 ▶); Mustafa *et al.* (2012 ▶); Scozzafava *et al.* (2003 ▶); Bouissane *et al.* (2006 ▶). For similar compounds see: Abbassi *et al.* (2012 ▶, 2013 ▶); Chicha *et al.* (2014 ▶).
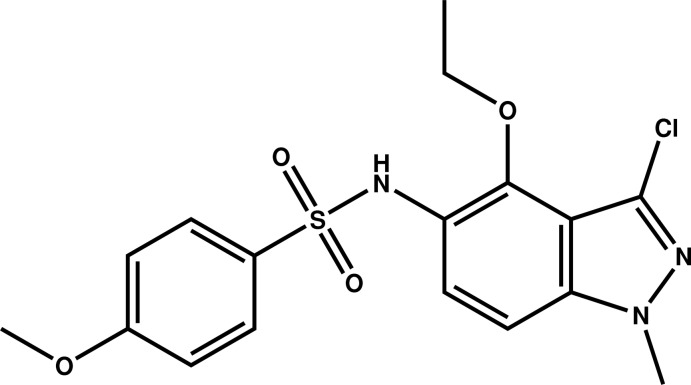


## Experimental   

### 

#### Crystal data  

C_17_H_18_ClN_3_O_4_S
*M* *_r_* = 395.85
Triclinic, 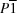

*a* = 8.5296 (9) Å
*b* = 8.6165 (9) Å
*c* = 12.9821 (14) Å
α = 91.810 (6)°
β = 102.566 (5)°
γ = 100.514 (5)°
*V* = 913.10 (17) Å^3^
*Z* = 2
Mo *K*α radiation
μ = 0.35 mm^−1^
*T* = 296 K
0.40 × 0.36 × 0.31 mm

#### Data collection  

Bruker X8 APEX diffractometer
Absorption correction: multi-scan (*SADABS*; Bruker, 2009 ▶) *T*_min_ = 0.693, *T*_max_ = 0.747
20149 measured reflections
4353 independent reflections
3825 reflections with *I* > 2σ(*I*)
*R*_int_ = 0.022

#### Refinement  

*R*[*F* ^2^ > 2σ(*F* ^2^)] = 0.044
*wR*(*F* ^2^) = 0.128
*S* = 1.04
4353 reflections
235 parameters
H-atom parameters constrained
Δρ_max_ = 0.51 e Å^−3^
Δρ_min_ = −0.62 e Å^−3^

### 

Data collection: *APEX2* (Bruker, 2009 ▶); cell refinement: *SAINT* (Bruker, 2009 ▶); data reduction: *SAINT*; program(s) used to solve structure: *SHELXS97* (Sheldrick, 2008 ▶); program(s) used to refine structure: *SHELXL97* (Sheldrick, 2008 ▶); molecular graphics: *ORTEP-3 for Windows* (Farrugia, 2012 ▶); software used to prepare material for publication: *PLATON* (Spek, 2009 ▶) and *publCIF* (Westrip, 2010 ▶).

## Supplementary Material

Crystal structure: contains datablock(s) I. DOI: 10.1107/S1600536814010800/rz5125sup1.cif

Structure factors: contains datablock(s) I. DOI: 10.1107/S1600536814010800/rz5125Isup2.hkl

Supporting information file. DOI: 10.1107/S1600536814010800/rz5125Isup3.cml

CCDC reference: 1002205

Additional supporting information:  crystallographic information; 3D view; checkCIF report

## Figures and Tables

**Table 1:** Hydrogen-bond geometry (Å, °)

*D*—H⋯*A*	*D*—H	H⋯*A*	*D*⋯*A*	*D*—H⋯*A*
N3—H3*N*⋯O3^i^	0.86	2.13	2.974 (2)	169

Symmetry code: (i) 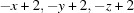.

**Fig. 1.**
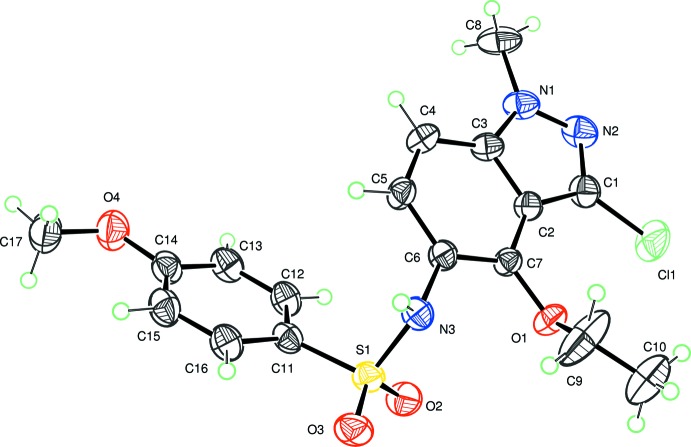
The molecular structure of the title compound with displacement ellipsoids drawn at the 50% probability level. H atoms are represented as small circles.

**Fig. 2.**
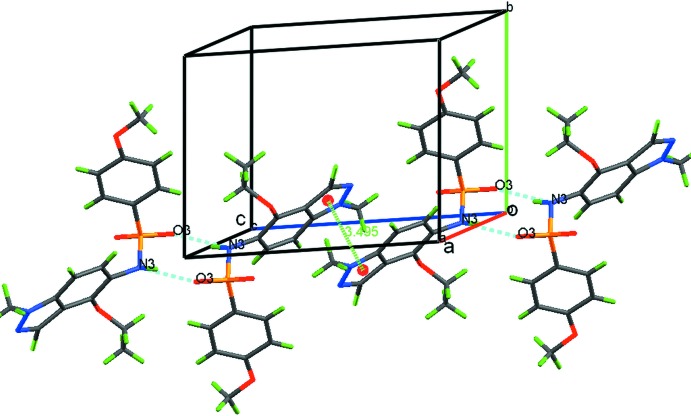
Partial crystal packing of the title compound, showing molecules linked by hydrogen bonds (blue dashed lines) and forming dimers linked by π–π interaction (green dotted line).

## supplementary crystallographic information

### 1. Comment

Sulfonamides constitute an important class of drugs possessing various types of pharmacological activities such as antibacterial, anti-carbonic anhydrase, high-ceiling diuretic, hypoglycemic, antithyroid, anti-inflammatory, and antiglaucoma activities (El-Sayed *et al.*, 2011; Mustafa *et al.*, 2012; Scozzafava *et al.*, 2003; Bouissane *et al.*, 2006). Recently, some *N*-[7(6)-indazolyl]arylsulfonamides prepared by our research group showed important antiproliferative activity against some human and murine cell lines (Abbassi *et al.*, 2012; Abbassi *et al.*, 2013; Chicha *et al.*, 2014).

The molecule of the title compound (Fig. 1) is built up from two fused five- and six-membered rings (N1/N2/C1–C7) almost coplanar, with a maximum deviation of 0.020 (2) Å for atom C2. The dihedral angles between the indazole ring system and the mean plane through the benzene ring and the 4-ethoxy-1-methyl group are 32.22 (8) and 57.5 (3)°, respectively. The cohesion of the crystal structure is ensured by N3–H3N···O3 hydrogen bonds (Table 1) between centrosymmetric molecules forming dimers, which are linked by π–π interactions between diazole rings [intercentroid distance = 3.4946 (11) Å] into chains parallel to the [1 0 1] direction (Fig. 2).

### 2. Experimental

A mixture of 3-chloro-1-methyl-5-nitroindazole (1.22 mmol) and anhydrous SnCl_2_ (1.1 g, 6.1 mmol) in 25 mL of absolute ethanol was heated at 333 K for 6 h. After reduction, the starting material disappeared, and the solution was allowed to cool down. The pH was made slightly basic (pH 7–8) by addition of 5% aqueous potassium bicarbonate before extraction with ethyl acetate. The organic phase was washed with brine and dried over magnesium sulfate. The solvent was removed to afford the amine, which was immediately dissolved in pyridine (5 ml) and then reacted with 4-metoxybenzenesulfonyl chloride (1.25 mmol) at room temperature for 24 h. After the reaction mixture was concentrated *in vacuo*, the resulting residue was purified by flash chromatography (eluted with an ethyl acetate/hexane mixture 1:4 *v*/*v*). The title compound was recrystallized from ethanol (yield: 45%, m. p.: 382 K).

### 3. Refinement

H atoms were located in a difference Fourier map and treated as riding with C–H = 0.93–0.97 Å, N–H = 0.86 Å, and with *U*_iso_(H) = 1.2 *U*_eq_(C, N) or 1.5 *U*_eq_(C) for methyl H atoms. Two reflections (010 and 001) were removed from the last cycles of refinement because affected by the beam stop.

### Crystal data

**Table:**

C_17_H_18_ClN_3_O_4_S	*Z* = 2
*M**_r_* = 395.85	*F*(000) = 412
Triclinic, *P*1	*D*_x_ = 1.440 Mg m^−^^3^
Hall symbol: -P 1	Melting point: 382 K
*a* = 8.5296 (9) Å	Mo *K*α radiation, λ = 0.71073 Å
*b* = 8.6165 (9) Å	Cell parameters from 4353 reflections
*c* = 12.9821 (14) Å	θ = 2.5–27.9°
α = 91.810 (6)°	µ = 0.35 mm^−^^1^
β = 102.566 (5)°	*T* = 296 K
γ = 100.514 (5)°	Block, colourless
*V* = 913.10 (17) Å^3^	0.40 × 0.36 × 0.31 mm

### Data collection

**Table:**

Bruker X8 APEX Diffractometer	4353 independent reflections
Radiation source: fine-focus sealed tube	3825 reflections with *I* > 2σ(*I*)
Graphite monochromator	*R*_int_ = 0.022
φ and ω scans	θ_max_ = 27.9°, θ_min_ = 2.5°
Absorption correction: multi-scan (*SADABS*; Bruker, 2009)	*h* = −11→11
*T*_min_ = 0.693, *T*_max_ = 0.747	*k* = −11→11
20149 measured reflections	*l* = −17→17

### Refinement

**Table:**

Refinement on *F*^2^	Primary atom site location: structure-invariant direct methods
Least-squares matrix: full	Secondary atom site location: difference Fourier map
*R*[*F*^2^ > 2σ(*F*^2^)] = 0.044	Hydrogen site location: inferred from neighbouring sites
*wR*(*F*^2^) = 0.128	H-atom parameters constrained
*S* = 1.04	*w* = 1/[σ^2^(*F*_o_^2^) + (0.0649*P*)^2^ + 0.4317*P*] where *P* = (*F*_o_^2^ + 2*F*_c_^2^)/3
4353 reflections	(Δ/σ)_max_ = 0.001
235 parameters	Δρ_max_ = 0.51 e Å^−^^3^
0 restraints	Δρ_min_ = −0.62 e Å^−^^3^

### Special details

**Table:**

Geometry. All e.s.d.'s (except the e.s.d. in the dihedral angle between two l.s. planes) are estimated using the full covariance matrix. The cell e.s.d.'s are taken into account individually in the estimation of e.s.d.'s in distances, angles and torsion angles; correlations between e.s.d.'s in cell parameters are only used when they are defined by crystal symmetry. An approximate (isotropic) treatment of cell e.s.d.'s is used for estimating e.s.d.'s involving l.s. planes.
Refinement. Refinement of *F*^2^ against all reflections. The weighted *R*-factor *wR* and goodness of fit *S* are based on *F*^2^, conventional *R*-factors *R* are based on *F*, with *F* set to zero for negative *F*^2^. The threshold expression of *F*^2^ > σ(*F*^2^) is used only for calculating *R*-factors(gt) *etc*. and is not relevant to the choice of reflections for refinement. *R*-factors based on *F*^2^ are statistically about twice as large as those based on *F*, and *R*- factors based on all data will be even larger.

### Fractional atomic coordinates and isotropic or equivalent isotropic displacement parameters (Å^2^)

**Table:**

	*x*	*y*	*z*	*U*_iso_*/*U*_eq_	
C1	0.5956 (2)	1.2540 (2)	0.53435 (14)	0.0465 (4)	
C2	0.6304 (2)	1.16538 (19)	0.62328 (12)	0.0362 (3)	
C3	0.4744 (2)	1.0918 (2)	0.63444 (13)	0.0398 (4)	
C4	0.4514 (2)	0.9921 (2)	0.71502 (15)	0.0442 (4)	
H4	0.3477	0.9433	0.7215	0.053*	
C5	0.5905 (2)	0.9706 (2)	0.78353 (14)	0.0415 (4)	
H5	0.5805	0.9040	0.8377	0.050*	
C6	0.7494 (2)	1.04510 (19)	0.77578 (12)	0.0360 (3)	
C7	0.77174 (19)	1.14377 (19)	0.69596 (12)	0.0345 (3)	
C8	0.1867 (2)	1.0920 (3)	0.53266 (18)	0.0650 (6)	
H8A	0.1396	1.1448	0.4730	0.098*	
H8B	0.1465	1.1199	0.5929	0.098*	
H8C	0.1567	0.9795	0.5164	0.098*	
C9	1.0146 (4)	1.3349 (4)	0.7609 (3)	0.1024 (13)	
H9A	1.0714	1.2835	0.8190	0.123*	
H9B	0.9409	1.3902	0.7881	0.123*	
C10	1.1304 (4)	1.4471 (4)	0.7272 (3)	0.0911 (10)	
H10A	1.1881	1.5222	0.7854	0.137*	
H10B	1.0757	1.5013	0.6710	0.137*	
H10C	1.2066	1.3947	0.7023	0.137*	
C11	0.8443 (2)	0.6987 (2)	0.84226 (13)	0.0404 (4)	
C12	0.7169 (2)	0.6368 (2)	0.75581 (14)	0.0463 (4)	
H12	0.7098	0.6828	0.6915	0.056*	
C13	0.6019 (3)	0.5074 (2)	0.76605 (16)	0.0497 (4)	
H13	0.5163	0.4665	0.7087	0.060*	
C14	0.6129 (2)	0.4373 (2)	0.86199 (16)	0.0468 (4)	
C15	0.7421 (3)	0.4963 (2)	0.94719 (15)	0.0517 (5)	
H15	0.7518	0.4475	1.0106	0.062*	
C16	0.8564 (2)	0.6281 (2)	0.93729 (14)	0.0477 (4)	
H16	0.9417	0.6694	0.9947	0.057*	
C17	0.4980 (3)	0.2324 (3)	0.9591 (2)	0.0686 (6)	
H17A	0.4045	0.1478	0.9498	0.103*	
H17B	0.4982	0.3051	1.0167	0.103*	
H17C	0.5963	0.1898	0.9744	0.103*	
N1	0.36396 (19)	1.1398 (2)	0.55635 (13)	0.0501 (4)	
N2	0.4375 (2)	1.2384 (2)	0.49460 (13)	0.0539 (4)	
N3	0.89007 (18)	1.02121 (17)	0.85304 (11)	0.0404 (3)	
H3N	0.8863	1.0326	0.9184	0.048*	
O1	0.92080 (15)	1.21799 (16)	0.68322 (10)	0.0452 (3)	
O2	1.01006 (18)	0.87771 (18)	0.73054 (11)	0.0539 (3)	
O3	1.11721 (16)	0.89209 (18)	0.92468 (11)	0.0527 (3)	
O4	0.4905 (2)	0.31318 (18)	0.86454 (14)	0.0643 (4)	
S1	0.98089 (5)	0.87464 (5)	0.83452 (3)	0.04091 (13)	
Cl1	0.73113 (8)	1.37072 (8)	0.47676 (5)	0.0764 (2)	

### Atomic displacement parameters (Å^2^)

**Table:**

	*U*^11^	*U*^22^	*U*^33^	*U*^12^	*U*^13^	*U*^23^
C1	0.0518 (10)	0.0536 (10)	0.0352 (8)	0.0137 (8)	0.0083 (7)	0.0118 (7)
C2	0.0383 (8)	0.0405 (8)	0.0305 (7)	0.0087 (6)	0.0077 (6)	0.0051 (6)
C3	0.0344 (8)	0.0495 (9)	0.0344 (8)	0.0085 (7)	0.0054 (6)	−0.0009 (7)
C4	0.0357 (8)	0.0528 (10)	0.0435 (9)	0.0017 (7)	0.0138 (7)	0.0030 (7)
C5	0.0444 (9)	0.0443 (9)	0.0377 (8)	0.0052 (7)	0.0150 (7)	0.0100 (7)
C6	0.0370 (8)	0.0391 (8)	0.0317 (7)	0.0079 (6)	0.0062 (6)	0.0061 (6)
C7	0.0335 (7)	0.0380 (8)	0.0317 (7)	0.0048 (6)	0.0086 (6)	0.0046 (6)
C8	0.0363 (10)	0.1031 (18)	0.0536 (12)	0.0190 (11)	0.0023 (8)	−0.0030 (12)
C9	0.098 (2)	0.100 (2)	0.093 (2)	−0.0537 (18)	0.0542 (18)	−0.0367 (17)
C10	0.0769 (18)	0.090 (2)	0.093 (2)	−0.0280 (15)	0.0291 (16)	0.0033 (16)
C11	0.0454 (9)	0.0436 (9)	0.0341 (8)	0.0157 (7)	0.0066 (7)	0.0074 (7)
C12	0.0552 (10)	0.0470 (9)	0.0354 (8)	0.0141 (8)	0.0034 (7)	0.0070 (7)
C13	0.0547 (11)	0.0460 (10)	0.0440 (10)	0.0123 (8)	0.0002 (8)	0.0001 (8)
C14	0.0537 (10)	0.0386 (9)	0.0518 (10)	0.0150 (8)	0.0144 (8)	0.0051 (7)
C15	0.0646 (12)	0.0528 (11)	0.0407 (9)	0.0172 (9)	0.0118 (8)	0.0139 (8)
C16	0.0540 (10)	0.0529 (10)	0.0344 (8)	0.0141 (8)	0.0023 (7)	0.0088 (7)
C17	0.0824 (16)	0.0540 (12)	0.0782 (16)	0.0135 (11)	0.0346 (13)	0.0184 (11)
N1	0.0383 (8)	0.0712 (11)	0.0393 (8)	0.0152 (7)	0.0021 (6)	0.0049 (7)
N2	0.0538 (9)	0.0712 (11)	0.0379 (8)	0.0222 (8)	0.0034 (7)	0.0104 (7)
N3	0.0430 (8)	0.0459 (8)	0.0309 (7)	0.0105 (6)	0.0034 (6)	0.0074 (6)
O1	0.0373 (6)	0.0556 (7)	0.0418 (7)	0.0006 (5)	0.0133 (5)	0.0069 (5)
O2	0.0551 (8)	0.0700 (9)	0.0422 (7)	0.0175 (7)	0.0175 (6)	0.0119 (6)
O3	0.0402 (7)	0.0672 (9)	0.0472 (7)	0.0139 (6)	−0.0014 (6)	0.0099 (6)
O4	0.0689 (10)	0.0500 (8)	0.0710 (10)	0.0045 (7)	0.0145 (8)	0.0114 (7)
S1	0.0376 (2)	0.0510 (3)	0.0343 (2)	0.01182 (17)	0.00498 (16)	0.00904 (17)
Cl1	0.0761 (4)	0.0903 (4)	0.0633 (4)	0.0073 (3)	0.0187 (3)	0.0442 (3)

### Geometric parameters (Å, º)

**Table:**

C1—N2	1.315 (3)	C10—H10B	0.9600
C1—C2	1.412 (2)	C10—H10C	0.9600
C1—Cl1	1.705 (2)	C11—C16	1.385 (2)
C2—C3	1.406 (2)	C11—C12	1.395 (3)
C2—C7	1.406 (2)	C11—S1	1.7552 (19)
C3—N1	1.356 (2)	C12—C13	1.374 (3)
C3—C4	1.399 (3)	C12—H12	0.9300
C4—C5	1.366 (3)	C13—C14	1.394 (3)
C4—H4	0.9300	C13—H13	0.9300
C5—C6	1.416 (2)	C14—O4	1.358 (3)
C5—H5	0.9300	C14—C15	1.388 (3)
C6—C7	1.384 (2)	C15—C16	1.384 (3)
C6—N3	1.438 (2)	C15—H15	0.9300
C7—O1	1.3618 (19)	C16—H16	0.9300
C8—N1	1.454 (3)	C17—O4	1.425 (3)
C8—H8A	0.9600	C17—H17A	0.9600
C8—H8B	0.9600	C17—H17B	0.9600
C8—H8C	0.9600	C17—H17C	0.9600
C9—O1	1.401 (3)	N1—N2	1.351 (2)
C9—C10	1.403 (3)	N3—S1	1.6346 (15)
C9—H9A	0.9700	N3—H3N	0.8599
C9—H9B	0.9700	O2—S1	1.4249 (14)
C10—H10A	0.9600	O3—S1	1.4414 (13)
			
N2—C1—C2	112.46 (17)	C16—C11—C12	120.00 (18)
N2—C1—Cl1	119.64 (14)	C16—C11—S1	119.51 (14)
C2—C1—Cl1	127.89 (15)	C12—C11—S1	120.31 (13)
C3—C2—C7	120.42 (15)	C13—C12—C11	119.71 (17)
C3—C2—C1	103.30 (15)	C13—C12—H12	120.1
C7—C2—C1	136.23 (16)	C11—C12—H12	120.1
N1—C3—C4	130.62 (17)	C12—C13—C14	120.31 (18)
N1—C3—C2	106.77 (16)	C12—C13—H13	119.8
C4—C3—C2	122.60 (16)	C14—C13—H13	119.8
C5—C4—C3	115.92 (16)	O4—C14—C15	124.41 (18)
C5—C4—H4	122.0	O4—C14—C13	115.58 (18)
C3—C4—H4	122.0	C15—C14—C13	120.01 (18)
C4—C5—C6	122.96 (16)	C16—C15—C14	119.60 (17)
C4—C5—H5	118.5	C16—C15—H15	120.2
C6—C5—H5	118.5	C14—C15—H15	120.2
C7—C6—C5	120.98 (15)	C15—C16—C11	120.33 (18)
C7—C6—N3	119.18 (15)	C15—C16—H16	119.8
C5—C6—N3	119.83 (14)	C11—C16—H16	119.8
O1—C7—C6	124.09 (15)	O4—C17—H17A	109.5
O1—C7—C2	118.80 (14)	O4—C17—H17B	109.5
C6—C7—C2	117.09 (14)	H17A—C17—H17B	109.5
N1—C8—H8A	109.5	O4—C17—H17C	109.5
N1—C8—H8B	109.5	H17A—C17—H17C	109.5
H8A—C8—H8B	109.5	H17B—C17—H17C	109.5
N1—C8—H8C	109.5	N2—N1—C3	111.91 (15)
H8A—C8—H8C	109.5	N2—N1—C8	120.44 (17)
H8B—C8—H8C	109.5	C3—N1—C8	127.59 (19)
O1—C9—C10	115.4 (3)	C1—N2—N1	105.56 (15)
O1—C9—H9A	108.4	C6—N3—S1	120.30 (12)
C10—C9—H9A	108.4	C6—N3—H3N	116.8
O1—C9—H9B	108.4	S1—N3—H3N	110.2
C10—C9—H9B	108.4	C7—O1—C9	118.21 (16)
H9A—C9—H9B	107.5	C14—O4—C17	118.35 (19)
C9—C10—H10A	109.5	O2—S1—O3	119.64 (9)
C9—C10—H10B	109.5	O2—S1—N3	108.12 (8)
H10A—C10—H10B	109.5	O3—S1—N3	104.44 (8)
C9—C10—H10C	109.5	O2—S1—C11	108.74 (9)
H10A—C10—H10C	109.5	O3—S1—C11	108.06 (8)
H10B—C10—H10C	109.5	N3—S1—C11	107.20 (8)

### Hydrogen-bond geometry (Å, º)

**Table:**

*D*—H···*A*	*D*—H	H···*A*	*D*···*A*	*D*—H···*A*
N3—H3*N*···O3^i^	0.86	2.13	2.974 (2)	169

Symmetry code: (i) −*x*+2, −*y*+2, −*z*+2.
